# Spontaneous infarction within a meningioma with negative DWI: an imaging pattern in patients with acute neurological deterioration

**DOI:** 10.1259/bjrcr.20150039

**Published:** 2015-05-15

**Authors:** J Hall, Y Y Wang, P Smith, T Sutherland

**Affiliations:** ^1^Department of Neurosurgery, St Vincent’s Hospital, Melbourne, VIC, Australia; ^2^Department of Surgery, The University of Melbourne, St Vincent’s Hospital, Melbourne, VIC, Australia; ^3^Department of Radiology, St Vincent’s Hospital, Melbourne, VIC, Australia

## Abstract

Despite being slow growing and presenting with insidious symptoms, patients with a meningioma can have rapid neurological deterioration as a result of increased intracranial pressure (ICP). The cause of raised ICP is often the development of peritumoral oedema, although the mechanism remains poorly understood. Infarction of meningiomas has been reported. The authors report a series of two cases in which spontaneous meningioma infarction and the development of peritumoral oedema resulted in increased ICP, neurological deterioration and presentation.

Meningiomas are primary central nervous system (CNS) tumours that originate from the arachnoid cap cells, which form the outer layer of the arachnoid mater covering the brain and spine. They account for approximately 30% of adult primary CNS tumours^1^ and can be graded with World Health Organization (WHO) grades I–III. The vast majority of these tumours are grade I.^1^ Often these tumours are slow growing and therefore detected incidentally or with insidious symptoms, such as new-onset and slowly progressing headache and neurological deficits of psychological disturbance^2,3^ depending on the location of the lesion. However, the most common presenting complaint is focal or generalized seizures, the pathological basis of which is poorly understood, but is likely be related to the location of the lesion, and surrounding oedema.^2,3^ There have, however, been several case reports of acute presentation and rapid symptomatic deterioration of patients with meningioma after presumed infarction of the tumour.^4–8^ We believe this is the first single-centre case series of two such patients to present acutely following infarction of a meningioma.

## Case report

### Case 1

**Figure 1.**
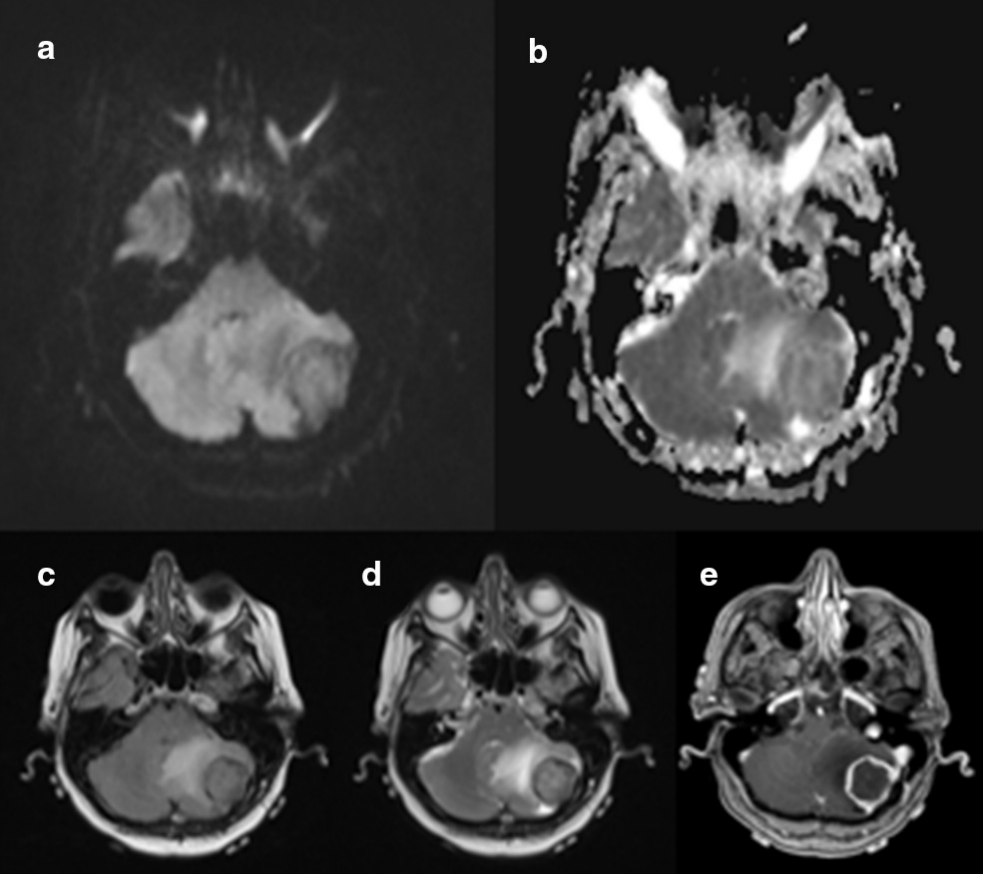
MRI scan of Case 1 demonstrating an extra-axial well-circumscribed mass within the left posterior fossa with a dural base against the left occipital bone. Diffusion-weighted image (DWI) (a) and associated apparent diffusion coefficientimage (b) demonstrate no restriction in diffusion. Fluid attenuation inversion recovery (c) and a *T*_2_ weighted image (d) shows associated increased signal consistent with peritumoral oedema and subsequent mass effect and effacement of the fourth ventricle. Contrast enhanced *T*_1_ image (e) shows a marginally enhancing lesion with dural tail.

A 68-year-old female presented 4 days following the onset of nausea, headache and neck pain. Her symptoms were increasing in severity and associated with left-sided inco-ordination and subtle facial weakness. A CT scan was performed and demonstrated a 3-cm left extra-axial posterior fossa lesion abutting the tentorium suggestive of a meningioma and associated with significant cerebellar oedema but no hydrocephalus. As a result, an MRI of the brain was performed that demonstrated a lesion with marginal enhancement, causing adjacent significant oedema. Diffusion-weighted imaging (DWI) demonstrated no restriction in diffusion and therefore was suggestive of necrosis (Figure 1). The patient underwent a left posterior fossa craniotomy and excision of the lesion. Histopathology confirmed a fibroblastic meningioma, WHO grade I with extensive infarct-type necrosis but no other atypical features. She recovered well from the procedure and was discharged home. At her 3-month review, the patient had made a full recovery, with resolution of her presenting symptoms and no signs of cerebellar dysfunction. Repeat MRI showed no evidence of recurrent tumour.

### Case 2

**Figure 2.**
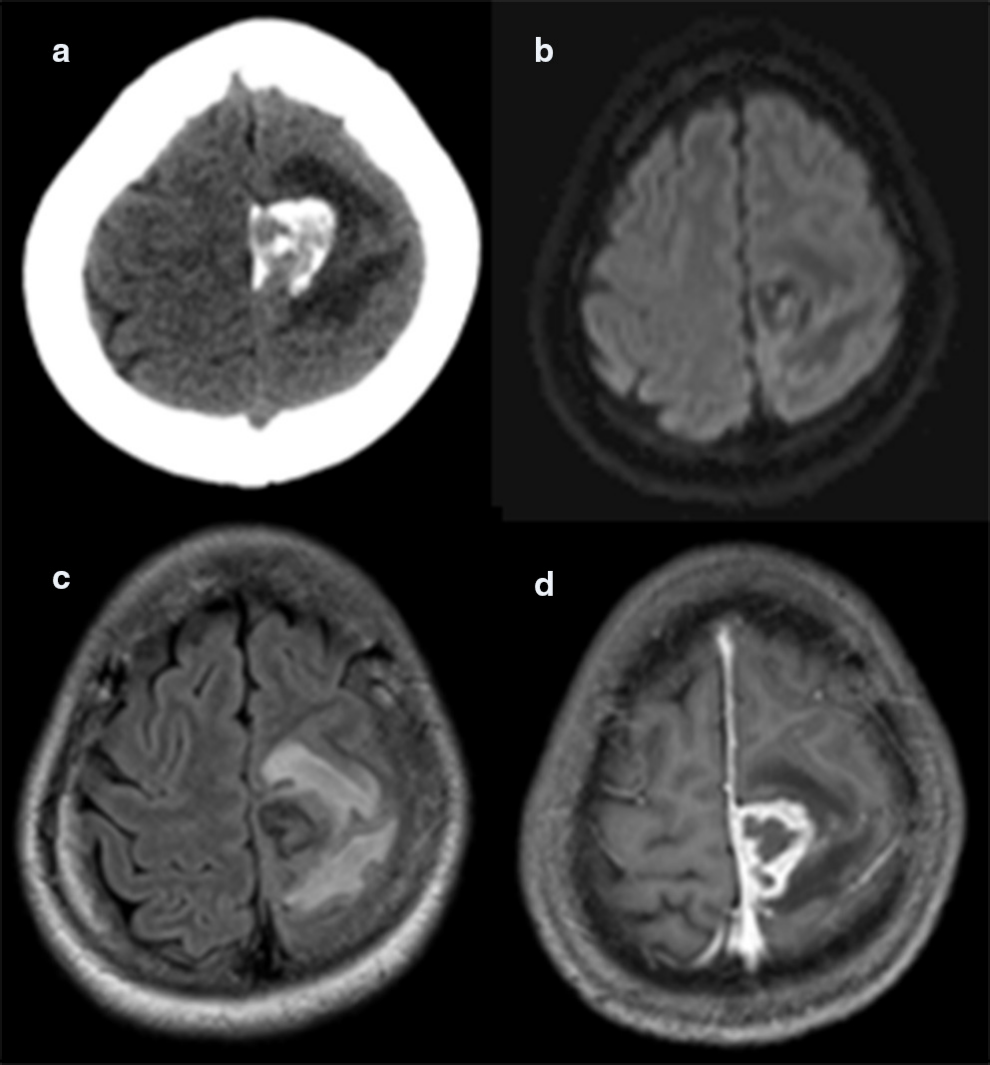
MRI and CT image of Case 2 demonstrating a left extra-axial parafalcine mass compressing the pre- and postcentral gyri and abutting the paracentral lobule. The CT image (a) shows heavy calcification and associated oedema. Peritumoral oedema is further demonstrated on the fluid attenuation inversion recovery image (c) with no diffusion restriction (b). The contrast-enhanced *T*1 weighted image (d) shows a marginally enhancing parafalcine mass with associated dural tail.

A 34-year-old female was transferred from a peripheral hospital 10 days after an elective lower uterine section caesarean section for a breech presentation for which she had an epidural anaesthetic. She presented to her general practitioner with a 2-day history of increasing right leg weakness. She was referred to her local hospital where she presented as no longer able to walk. Her examination demonstrated 0/5 power in right ankle flexion, extension, eversion and inversion with increased tone. Her cranial nerve examination was normal as was her tone and reflexes. An urgent MRI of the spine was undertaken that revealed no evidence of epidural collection or neural compression. She subsequently underwent a CT scan of the brain that demonstrated a dural-based lobulated, heavily calcified left parafalcine mass associated with extensive oedema consistent with a meningioma (Figure 2). The bulk of the calcification was at the periphery of the lesion with relative sparing of the central elements. 8-mg dexamethasone was administered intravenously prior to transfer to a tertiary centre where an MRI of the brain was undertaken. This showed a left extra-axial parafalcine mass compressing the pre- and postcentral gyri and abutting the paracentral lobule. It was isointense to cortex on *T*_2_ weighted images with hypointense peripheral component corresponding to areas of calcification. The mass demonstrated peripheral enhancement and an enhancing dural tail. There was no evidence of restricted diffusion. Overall, it was felt most likely to be a meningioma with some atypical features. She underwent a left frontoparietal craniotomy and resection of the lesion. Histology demonstrated a meningothelial meningioma, WHO grade I with focal areas of necrosis and features, raising the possibility of embolization-related changes. She recovered well and was discharged home after brief inpatient rehabilitation. At her 4-month review, she was walking with 5/5 power in hip and knee flexion and extension and 4/5 power in ankle dorsiflexion and plantaflexion. A 6-month follow-up MRI showed no recurrence.

## Discussion

Meningiomas have an estimated prevalence of 97.5/100,000^9^ and are slow-growing CNS tumours. Meningiomas demonstrate a female predominance in the order of 2 : 1 and have a biphasic peak at the age of presentation in prepubescent children and the elderly. However, despite this predominance, advances in radiological imaging and the increasing ease of access have led to meningioma’s being discovered in asymptomatic individuals of any age. Risk factors for meningioma include ionizing radiation, hormones, family history and hereditary syndromes such as neurofibromatosis Type 2, although these remain contentious.^9^

As with all intracranial mass lesions, the presenting complaint varies depending on the location. However, owing to the overall slow growth rate of meningiomas, presenting symptoms are usually insidious in nature. A slowly progressing headache, progressive personality change, partial seizures or a monoparesis are the most common presenting symptoms.^3^ Meningiomas can occasionally present with more acute neurological symptoms, often owing to raised intracranial pressure (ICP). In the limited case reports documenting infarction of a meningioma,^4–7,10^ patients have presented with severe and worsening headache, nausea, vomiting, seizures or decreased level of consciousness.

Meningioma can infarct for several reasons and tumour necrosis, seen on pathology, is a sign of either embolization of the tumour or characteristic of higher-grade tumours. Penfield^11^ was the first to describe the peripheral vascular supply of meningiomas and believed the centre of the tumour to be a "watershed area". Since then, there have been case reports demonstrating infarction of a meningioma spontaneously^5^ or as a consequence of hypotension, intraoperatively^4^ or following myocardial infarction and cardiac arrest.^7^

The reason for meningiomas or infarcted meningiomas developing peritumoral oedema remains unclear. While aetiologies including tumour size, location, histological subtypes, vascular supply, level of prostaglandins and sex hormones have all been investigated, results have been disappointing with no clear aetiology emerging. Hou et al^12^ in their review describe four theories that have been suggested to explain the development of peritumoral oedema and the relationship of angiogenic factors on the development of oedema. Secretory–excretory phenomenon, in which meningiomas of various histological subtypes produce reactions that lead to perivascular proteinaceous substances being secreted and inducing oedema formation in the surrounding brain tissue. The cerebral compressive theory suggests that the larger the tumour, the more compression of surrounding brain tissue, inducing ischaemia and subsequent cytotoxic oedema. The vascular compression theory, although not consistent with the case presented above, describes peritumoral oedema as a consequence of tumours that occlude major cerebral veins or sinuses. Finally, the hydrodynamic theory that states that peritumoral oedema occurs in the presence of intratumoral congestion. In the setting of a meningioma outgrowing its blood supply, several angiogenic factors are released and new but highly permeable blood vessels develop. Permeability of these vessels at the tumour–pial interface leads to peritumoral oedema through vasogenic oedema. In the setting of acute meningioma infarction, these mechanisms lead to the development of acute peritumoral oedema, rapid onset of symptoms and, as a result, little time for compensatory mechanisms to reduce ICP.

Most patients who present with new or acute deterioration of neurological symptoms will undergo a CT scan in the first instance and may subsequently undergo further imaging, including MRI. Findings on MRI for a typical meningioma include homogenous contrast enhancement, well-defined margins and a dural tail as they are extra-axial. CT scan may show calcifications that can be appreciated on MRI as blooming artefact on susceptibility weighted images. Peri et al^10^ demonstrated these features in the preoperative evaluation a patient with two meningiomas. Following diagnostic percutaneous angiography, in which they believe one of the meningiomas infarcted, they repeated an MRI scan. On MRI in the acute setting, this would result in elevated signal on DWI with a correspondingly low apparent diffusion coefficient (ADC) value, similar to infarction elsewhere. Filippi et al^13^ attempted to correlate the DWI with histopathological findings. They found that atypical and malignant meningiomas exhibited hyperintense signal on DWI and a decrease in the average diffusion constant calculated from the ADC maps. What they also described was 2/17 cases exhibited hypointensity on DWI, and iso- and hyperintensity on the ADC maps. Histology from these two patients exhibited areas of necrosis and central infarct. In the cases that we are presenting, we find no evidence of diffusion restriction, despite the MRIs being performed within a few days of symptom onset. Lack of diffusion restriction would be consistent with necrosis within the meningioma. We postulate that the infarction and subsequent necrosis leads to the development of peritumoral oedema through mechanisms previously described. The development of oedema and subsequent increase in the ICP is what leads to the development of neurological symptoms and presentation.

The management of a patient with an asymptomatic meningioma usually involves a watch and wait approach. In the case of a patient presenting with an infarcted meningioma, the initial management aim should be to stabilize the patient, treat presenting symptoms, and further evaluate with diagnostic imaging and then surgery. Steroids, while effective in reducing the ICP in patients with malignant intracranial tumours, are relatively ineffective in reducing peritumoral oedema associated with standard meningiomas.^12^ However, given the low morbidity associated with steroid use, they should still be employed in patients with a meningioma presenting with acute neurological deterioration. Ultimately, complete surgical resection of symptomatic lesions is desirable if anatomically favourable.

## Conclusions

We present a single-centre case series of patients with acute infarction of a meningioma. Infarction of meningioma is a rare cause of acute neurological deterioration in a previously stable patient. Evaluation with CT and MRI scan can assist in diagnosing the cause of the acute presentation and DWI is important in demonstrating restricted diffusion and infarction of the meningioma as a cause for the sudden neurological deterioration of a previously stable patient with meningioma. Despite this, our series demonstrates that the development of necrosis following infarction means that these patients do not exhibit the expected restriction on DWI. Infarction can lead to the development of or increase in vasogenic oedema that can contribute to the presentation and symptoms that patients experience. Finally, while steroids are the mainstay of treatment of malignant neoplasms to reduce oedema and subsequent ICP, they are relatively ineffective against meningioma-associated oedema and early surgical resection should be a priority.

## Learning points

1. Meningiomas, while slow growing and usually presenting with insidious onset of symptoms, can undergo acute changes leading to acute neurological deterioration.
2. Infarction of a meningioma is one such acute change that can lead to neurological deterioration.
3. Infarction and necrosis can lead to the development of peritumoral oedema and subsequent rise in ICP.
4. Restriction on DWI is useful for demonstrating infarction within tissues. However, meningiomas, which have infarcted and subsequently developed necrosis, may demonstrate a lack of diffusion restriction.
